# Rapid Cure of a Disease Thus Far Known as Absolutely Fatal

**Published:** 1884-03

**Authors:** B. Fincke


### CLINICAL BUREAU.

### RAPID CURE OF A DISEASE THUS FAR KNOWN AS ABSOLUTELY FATAL.

(*Observed by Dr. Buchmann in Alvensleben, translated from the "Allg. Hom. Zeitung Leipzig," Vol. CVII, p. 22, by B. Fincke, M. D.*)

Whenever from time to time I shall offer a case in this journal, an elaborate model-report of the whole process of the disease should not be expected, since I can only make a note of the remedies given and must replenish the rest from memory. On the whole, I am of the opinion that short reports of cases—if only the striking healing action of a remedy is clearly shown—are more useful, for the actual cure of a disease is much more important for us than the description of its remarkable course.

The farmer D., fifty years old, phlegmatic, tall and slender, suffered during the past summer several times of irregular chills with following heat and loss of appetite, so that he feared the development of an intermittent; but he did not pay sufficient attention to it, since he was not obliged to interrupt his labors in the field permanently.

September 12th I was called to see him, because he felt himself too weak to leave his bed. Frequent shiverings, great muscular weakness, restless sleep, loss of appetite, tongue coated and inclined to dryness, language difficult, slight bronchial catarrh, increased temperature of the skin, though the pulse was not accelerated through the day; increased thirst, drawing pains at the thorax and in the abdomen, paleness—all these symptoms left no doubt that here was a case of *Typhus ambulatorius*,* localized as gastric and duodenal catarrh, since the sluggish evacuations were colored a grayish white. In the night, symptoms of decline and talking without sense.

Till end of September the condition—under the use of *Chelidonium majus*, 6 cent., and *Bryonia alba*, 6 cent., in globules—had so far changed that the tongue was moist and clean, the nightly delirium had ceased, and some spoonfuls of soup could be taken. The patient felt better, and, except a great sensation of weakness, had nothing to complain of.

Oct. 1st.—Patient complains, with a hoarse voice, of pains in the pharynx, which, accompanied by a wound-like sensation, extended down the throat as far as the posterior part of the sternum; of impossibility to swallow; of increased pains in the larynx on coughing; of retching, with ejection of much tough mucus; of great heat and much thirst. The pulse was accelerated. Urine transparent, of reddish color; tongue coated, thick, and grayish yellow. From half of the hard palate backward, uvula, tonsils, and pharynx were covered with a thick, grayish-white substance, which gradually entered into dispersed whitish granules, of poppy-seed size, toward the front of the palate.

Dr. Kafka says in his *Therapia* on this disease: "The thrush of the adult, which commonly extends into the pharynx and larynx, and even into the œsophagus, is incurable. It is a veritable bird of death which announces the approaching end. In order to avoid laying our hands in our laps, we cause the cavity of the mouth and throat to be painted with a linctus composed of *Borax* or *Sulphuric acid* and *Mel rosatum*; but the result is always—*Certa mors!*"

In spite of my terror at the appearance of this disease, the thought occurred to me that a remedy which frequently has caused speedy improvement in *diphtheritis* possibly might prove curative also in this infectious disease, localized in the same region, though thus far no cure had been published. I therefore prescribed ℞ *Mercur. cyan.*, 15 cent., about twenty globules, to

---

* According to Kafka, typhus is subdivided into the non-localized and localized typhus. The non-localized typhus, again, is either abortive typhus or typhus ambulatorius. The localized typhus comprises the ileotyphus or typhus abdominalis and the typhus exanthematicus or petechialis. The typhus ambulatorius has a tedious course—lasts often eight to twelve weeks—and terminates mostly in convalescence. But the recovery is doubtful when the disease turns into localized typhus, and the termination is absolutely fatal when localized upon the mucous membrane of the mouth and throat under the symptoms of muguet (severe form of thrush) and diphtheria.—B. F.

be dissolved in a cup of water, and the patient—in case he could not swallow—to take some of the solution every two hours and to keep it for a few minutes in his mouth.

The next morning the wife of the patient showed me a convoluted lump of tough mucus which could have filled more than a spoon, mixed throughout with white granules of poppy-seed size in great quantity, which on waking up in the morning had been expelled by the patient in one piece. The mucous membrane in the cavity of the mouth and throat appeared, with the exception of the tongue and gums, dark red, spongy, and only here and there covered with white granules. Much tough mucus had been expectorated; swallowing difficult; painful hoarseness; sensation of a wound, as before, down the throat; tongue clean, with woundlike pain at the edges; urine reddish yellow, turbid, with sediment of like color; skin perspiring—more so on the chest—œdema of the feet less.

℞ *Merc. cyan.*, 15 cent. u. s.

Oct. 3d.—No more granulues to be discovered in the mouth. Mucous membrane paler, less swollen. At the uvula and palate in two places aphthous ulcers. Cough worse with profuse expectoration of mucus.

℞ Apis. mel., 30 cent., three globules every three hours.

Oct. 7th.—Œdema pedum gone. Since day before yesterday toward 4 P. M., chill, lasting half an hour. Then heat till noon, then profuse perspiration till toward morning of next day. Tongue moist; clean. Stool, until now, only every third day and difficult. Urine of normal color with gray-red sediment.

℞ Ipec., 6 cent., three globules every three hours.

Oct. 10th.—The fever attack gradually disappeared.

℞ China, 3d dec., three times a day.

Oct. 15th.—Perspiration toward morning on the chest. Appetite indifferent. Pale skin and pale mucous membrane in the mouth.

℞ Pulsat., 6 cent., five globules every three hours. Meat soup and wine.

Oct. 20th.—Disappearance of night-sweats, quiet sleep, trying to walk, external appearance better.

℞ Ferr carb., 4 dec., about as much as a lentil three times a day.

Convalescence.

---

In this connection the following observation of Dr. B. Fincke may be interesting:

CASE OF DIPHTHERIA CURED BY BORAX 9C.

Mrs. H., sixty years old, robust workingwoman, New York. Nov. 29th, 1881, was taken with chilliness, fever, sensation of a lump in the throat or windpipe, that she can swallow only with great pains. She cannot speak; it is as if she had the whole mouth full of pap. She lies in bed since the 26th inst. When spitting she expels bloody mucus. Now the children had had thrush and got well on Borax v., 9c., in water, given by teaspoonful every two hours, and it happened that two powders were left unused. These patient took in the same way as the children did.

I saw her to-day at 10 A. M., and found her looking better than expected. Pulse 102. The whole pharynx was covered with a dirty yellow thick skin, which in some places became detached in flakes. The uvula only was free, and swollen with congested veins along its length. There was, however, no fœtor, no bad taste. But the nose felt wound-like in its upper part. On swallowing, cutting pains extending to both ears. Last night the two powders had given out, and so she took on her own account a dose of Bellad., 9c., from her medicine case. But she had a bad night of it. This morning, however, she was better. The mucus was expelled freely; she could not describe sufficiently how much there was of it; it was suffocating. Since Borax had acted so well with her, as also with the children, who had been reported to have thrush, but very likely also had had diphtheria, because as now is described to me, their tongues, cheeks, pharynxes—in fact, the whole cavities of their mouths and throats, were covered with a thick skin. I gave again ℞ Borax ven., 9c., in half a tumbler of water, a teaspoonful every two hours as before.

Dec. 5th.—The diphtheritic skin was all gone. Only the uvula looks livid and swollen from the enlarged veins along its length. Pains in throat at the left side on swallowing.

℞ Lachesis, 9c., in water, u. s.

Convalescence.

---

There seems to be a close relationship between the nosographical groups of thrush, muguet, diphtheria, and croup, having as *fundamentum divisionis* the exudation of an excrementitious matter, viz.: fibrine, which seems to go no deeper than the epithelium at first and affects the deeper tissues only in the later stages. This exudation forms the pseudomembranous covering of the mucous membrane and endangers life on account of its extension in all directions.

Hartmann in his *Kinderkrankheiten* already discriminates between common thrush and muguet, which he calls "*stomatitis diphtheritica*, a pseudomembranous form of the mucous membrane of the mouth and throat" (p. 128). Here in the latter case the thrush remedy, Borax (see *Chronic Diseases*, II, p. 290, symph. 150-152) cured diphtheria, and in Buchmann's case the diphtheria remedy, Mercurius cyanatus, did the same for stomatitis diphtheritica or muguet or aggravated thrush.

*Ceterum censeo, macrodosiam esse delendam.*

B. FINCKE, M. D.

